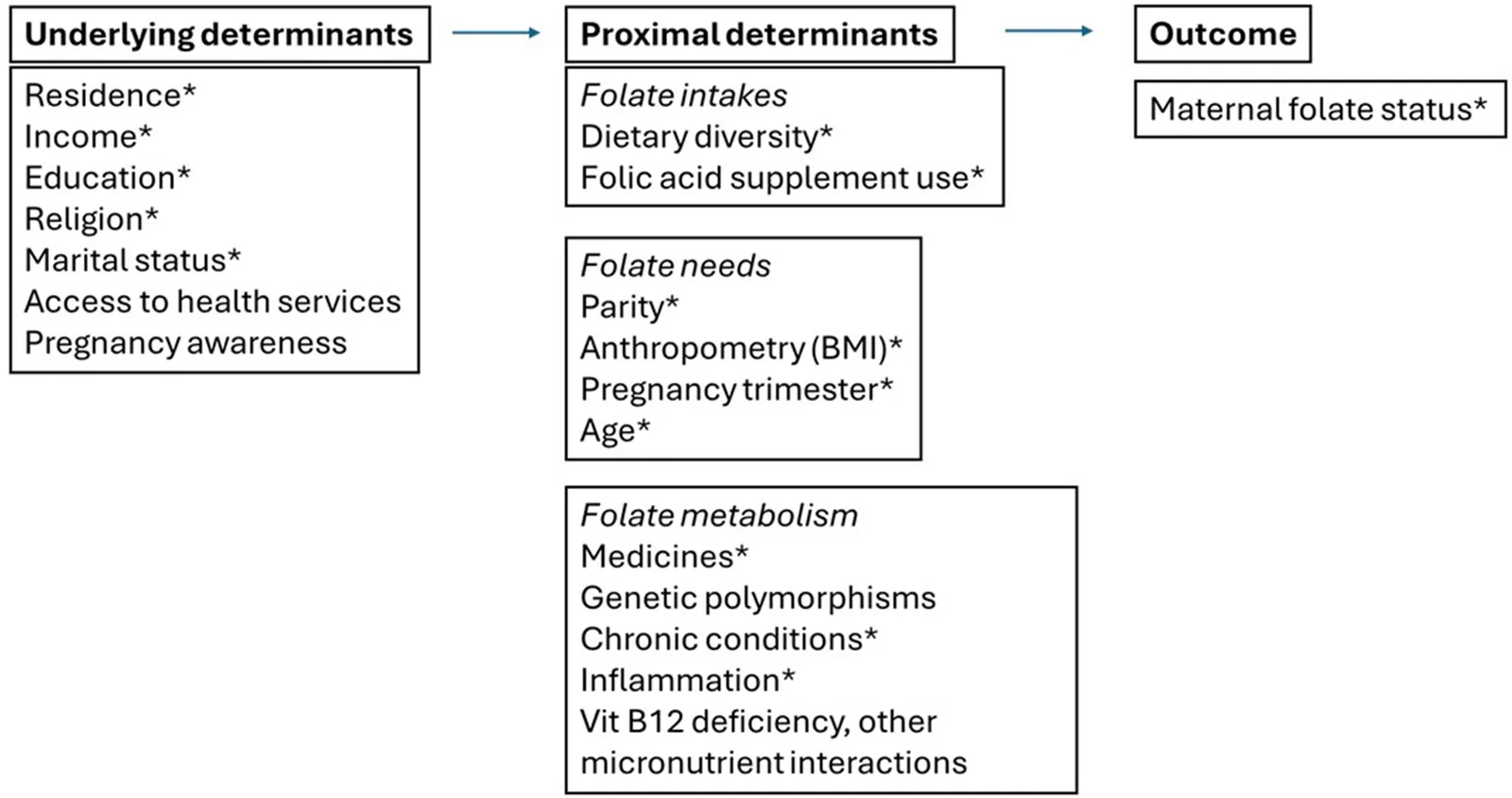# Prior Pregnancies Deplete Maternal Folate Status and Increase Risks of Neural Tube Defects in Addis Ababa, Ethiopia

**DOI:** 10.1016/j.tjnut.2026.101666

**Published:** 2026-06-10

**Authors:** Hasset Tamirat Molla, Edward JM Joy, Dawd Gashu, Demiraw Bikila, Tadesse Lejisa Garomsa, Barbara Stoecker, Fanny Sandalinas

**Affiliations:** 1 Center for Food Science, Addis Ababa University, Addis Ababa, Ethiopia; 2 Faculty of Epidemiology and Population Health, London School of Hygiene & Tropical Medicine, London, United Kingdom; 3 National Clinical Chemistry Reference Laboratory, Ethiopian Public Health Institute, Addis Ababa, Ethiopia; 4 Nutritional Science Department, Oklahoma State University, Stillwater, OK, United States

**Keywords:** folate, neural tube defects, pregnancy, parity, Ethiopia, antenatal care, folic acid supplementation

## Abstract

**Background:**

Folate is essential for fetal neural development, and maternal deficiency during early pregnancy increases risk of neural tube defects (NTDs). Ethiopia has among the highest NTD rates globally, yet data on folate status determinants among pregnant women are scarce.

**Objectives:**

This study aimed to identify the role of dietary and demographic factors in determining maternal folate status and to interpret these findings in the context of NTD prevention.

**Methods:**

We conducted a cross-sectional study among pregnant women (*n* = 404) attending their first antenatal care visit at 3 public health centers in Addis Ababa, Ethiopia. Serum folate was measured using electrochemiluminescence immunoassay. Increased risk of NTD was defined by serum folate <24.3 nmol/L. Sociodemographic, dietary, and clinical data were collected by questionnaire. We examined factors associated with serum folate concentrations using linear regression models.

**Results:**

A total of 61% of women had concentrations below levels estimated to be protective against NTDs. Supplement use was rare (<3%). After adjustment for wealth, residency, dietary intake, body mass index, inflammation and trimester, serum folate was lower among women with 2 (*β* = –2.2 nmol/L; *P* = 0.04), 3 (*β* = –6.2 nmol/L; *P* < 0.001), 4 (*β* = –11.2 nmol/L; *P* < 0.001), and 5 (*β* = –13.7 nmol/L; *P* < 0.001) prior pregnancies compared with primigravidae. Serum folate was higher in the second than the first trimester (*β* = 3.90 nmol/L; *P* = 0.003).

**Conclusions:**

Higher gravidity is strongly associated with lower folate status, consistent with maternal depletion. Multiparous women may be at increased risk of NTD-affected pregnancies. Interventions to improve folate status before conception, including supplementation, food fortification, and strategies to lengthen interpregnancy intervals, should be prioritized in Ethiopia.

## Introduction

Folate is a water-soluble B vitamin, essential throughout life, and particularly critical during early stages of human development [1]. Because of its critical role in DNA, RNA, and protein synthesis, folate inadequacy during pregnancy is associated with several adverse outcomes [1]. These include congenital malformations of the brain and spinal cord caused by failure of the neural tube to close between 21 and 28 d after conception, underscoring the importance of attaining adequate folate status among mothers before conception and in very early pregnancy. These neural tube defects (NTDs) comprise about one-tenth of the burden of all congenital conditions globally and constitute the third largest congenital burden after congenital heart disease and Down syndrome [2].

Ethiopia bears a particularly high burden of NTDs. A pooled meta-analysis estimated a national incidence of ∼71.5 NTDs per 10,000 births (95% confidence interval: 57.8, 86.6) [3], ∼7 times higher than the estimated prevalence in sub-Saharan Africa [3,4]. Madrid et al. [4] reported an under-5 mortality rate attributable to NTDs of 104 per 10,000 live births in Eastern Ethiopia, 4–25 times greater than rates observed at mortality surveillance sites in 8 other study countries. Despite the preventable nature of NTDs through adequate folate intake and supplementation before conception and in early pregnancy [1], low folate status remains widespread.

Global estimates indicate that folate deficiency is highly prevalent among nonpregnant women of reproductive age in most countries [5]. In the 2015 Ethiopian national micronutrient survey, 78% of nonpregnant adult women had red blood cell folate concentrations indicative of increased risk of NTD-affected pregnancies, with evidence of spatial variability in status linked to farming systems [6,7]. National estimates of the folate status of pregnant women are unavailable in Ethiopia; however, community-based studies consistently report a high prevalence of folate deficiency in pregnant women. In 2 studies in Eastern Ethiopia, nearly half of pregnant women were classified as folate deficient [8,9], whereas a hospital-based study in Addis Ababa found that 28% had red blood cell folate concentrations below the threshold (400 ng/mL) recommended for NTD prevention [10].

Most evidence on determinants of folate status during pregnancy originates from high-income settings, where the link between supplement use and folate status has been the primary focus. Studies conducted in Belgium and the Netherlands [11,12] have identified supplement use and socioeconomic status as major predictors of folate status during pregnancy. However, these findings may not be generalizable to low- and middle-income countries (LMICs), where dietary diversity is typically low, folic acid supplements are not widely available, and preconception care services are limited or underutilized. In Ethiopia, adherence to iron–folic acid supplementation programs varies widely, and uptake before conception is particularly low [13,14].

Understanding the maternal, socioeconomic, and dietary drivers of folate status in Ethiopia is therefore critical for informing the development of effective and equitable interventions to alleviate folate deficiency and avert NTD-affected births. The aim of this study was to identify the role of dietary and demographic factors in determining maternal folate status and to interpret these findings in the context of NTD prevention.

## Methods

### Setting

Public health centers in Addis Ababa, Ethiopia, were included in the study, as they are the primary entry point for antenatal care (ANC). The 10 city administrative subdivisions were listed, and 3 subcities (Nifas Silk Lafto, Addis Ketema, and Bole) were randomly selected, with 1 public health center per subcity chosen based on feasibility.

### Participants

#### Eligibility criteria

**FIGURE 1:**
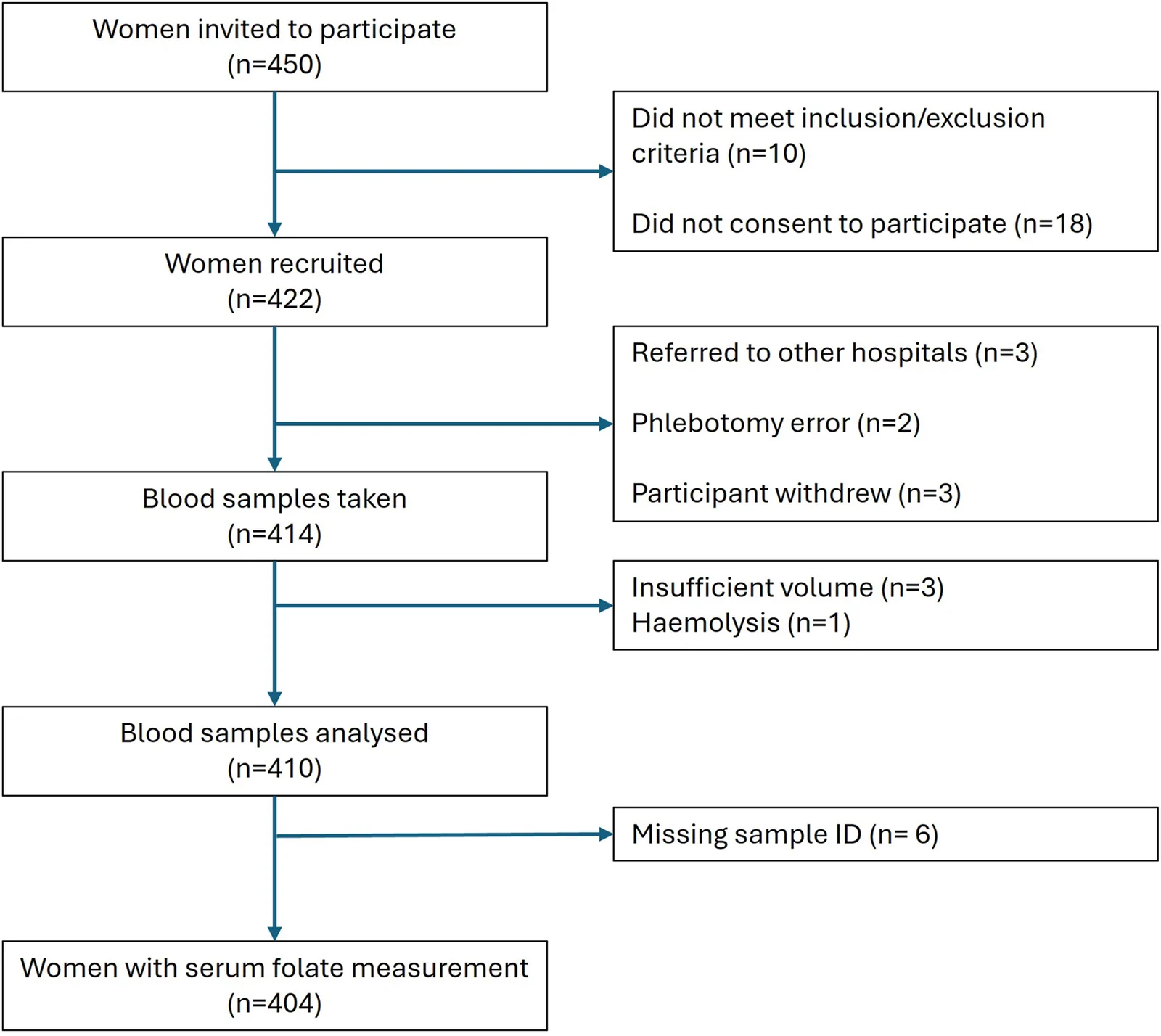
Participant flowchart.

Pregnant women were screened at ANC visits during April–August 2022. Women were included if attending their first ANC visit of the pregnancy during their first or second trimester. Women in their third trimester were excluded because the study focused on determinants of folate status in early pregnancy. Those seriously ill or hospitalized were excluded, including women with acute medical or obstetric conditions requiring inpatient care (e.g., severe infections, hypertensive disorders, major bleeding, complications requiring surgical intervention), or any condition impairing consent or safe participation. All eligible women attending the health centers were approached consecutively until the sample size was reached. Only those who provided written informed consent were enrolled (Figure 1).

### Sample size calculation

Sample size was estimated assuming a 49.3% prevalence of folate deficiency among pregnant women [8]. Statistical assumptions were a 95% confidence level, a 5% margin of error, and 80% power. The calculated sample size was 384, and a nonresponse rate was 10%, resulting in a target sample size of 422.

### Laboratory methods

#### Sampling procedure

A volume of 5 mL of venous blood was collected in serum separator tubes from nonfasting women by senior laboratory technicians working in the respective health centers. The samples were allowed to clot at room temperature and were centrifuged 30-60 min after sample collection, at 3 000 RPM for 10 minutes. Then, the serum was aliquoted into cryovial tubes. To avoid folate deterioration, the tubes were protected from light with aluminum foil and labeled in dim light. Samples were stored at –20°C in the laboratory of the respective health centers until transfer to the National Clinical Chemistry Reference Laboratory of Ethiopian Public Health Institute (EPHI) to be stored at –80°C until testing. Once aliquoted, the samples were frozen and thawed only once during laboratory testing.

#### Laboratory analysis

Serum folate concentrations were measured using the electrochemiluminescence method on a fully automated immunoassay analyzer (Roche/Hitachi Cobas 6000(e601) (Roche Cobas 6000 series) at the National Clinical Chemistry Reference Laboratory of EPHI using commercial kits (Roche). Two control samples [PreciControl Varia 1 (3.9 ng/mL) and PreciControl Varia Two (12 ng/mL), Roche] were analyzed for quality control. Quality control results were monitored over time using Levey–Jennings charts, and Westgard rules were applied to ensure that assay performance remained within acceptable limits.

Highly sensitive C-reactive protein (CRP) and α-1-acid glycoprotein (AGP) concentrations were analyzed using a fully automated clinical chemistry analyzer, Roche/Hitachi Cobas 6000 (c501) (Roche Cobas 6000 series).

### Dietary data

A diet diversity score was calculated based on the consumption of 10 food groups recalled over the 3 d before the interview. Additionally, a food frequency questionnaire was used to assess the weekly consumption frequencies of 20 food groups, including a group of folate-rich foods (supplementary text).

### Supplement data and other lifestyle factors likely to influence folate status

A questionnaire was administered to participants to assess supplement use before conception or in early pregnancy, substance use [including alcohol, tobacco, marijuana, and khat (*Catha edulis*)], and the use of medication known to interfere with folate metabolism (including clomiphene citrate for infertility and sodium valproate for epilepsy).

### Clinical data

Gestational age (GA) was assessed from a reliable date using the participants’ last normal menstrual period or early ultrasound. The data collectors also confirmed the GA from the medical chart on-site.

Gravidity was defined as the number of times a woman had been pregnant previously, regardless of the pregnancy outcome and GA. Primigravida was defined as first-time pregnant, multigravida as pregnant 2 times or more (including the current pregnancy).

### Ethical approval and consent for research participation

The parent study protocol received a favorable opinion from the Institutional Review Boards (IRBs) of Addis Ababa University (IRB # 04-14-2021) and Oklahoma State University (# IRB-24-234), and the National Research Ethics Review Board at the Ministry of Education, Ethiopia (IRB # 17/246/818/22). The protocol for this data analysis received a favorable opinion from the LSHTM Observational Research Ethics Committee (reference 32432, 19 June, 2025).

Before the study commencing, a formal letter seeking approval for the study was submitted to the Chief Executive Officer of each health center. In these letters, the purpose of the study and procedures of data collection together with the proposed participant information sheet were submitted, together with a request to access potential participants and, following their recruitment, access to their medical records from the ANC unit of the Gynecology and Obstetrics department. Subsequently, permission was granted by the Medical Director emphasizing the ethical conduct and regulatory compliance of the study. Recruitment to the study followed an informed consent process.

### Statistical analysis

The primary outcome was serum folate concentration (nmol/L), treated both as a continuous variable and as a binary indicator of increased risk of NTDs.

The WHO does not recommend a serum folate cut-off to predict NTD risk, as red blood cell folate is the preferred biomarker. However, recent modeling work has suggested that a serum folate concentration of 24.3 nmol/L is associated with optimal NTD prevention [15]. This threshold was therefore used as a reference for the interpretation of population-level folate adequacy.

A conceptual framework to support causal analysis was developed a priori based on plausible biological associations between exposures and maternal folate status, drawing on existing literature (Supplemental Figure 1). Thus, the exposure variables of interest included in the analysis were gravidity, GA (weeks), maternal age (years), BMI, use of supplements during preconception and early pregnancy, dietary diversity score, and reported consumption of folate-rich foods. Educational level, household income quintile, occupation, religion, and inflammatory markers (CRP and AGP) were included as potential confounders.

GA at enrollment was classified into trimesters: <14 wk (first) and 14–27 wk (second). A wealth variable was created by dividing monthly household income into quintiles. Educational attainment, occupation, and religion were treated as categorical variables.

We explored the data using histograms/boxplots and scatter plots of serum folate against each exposure. We estimated associations using linear regression, reporting *1*) unadjusted models and *2*) models adjusted for prespecified covariates. Model assumptions were examined through diagnostic plots, including linearity, homoscedasticity, and normality of residuals. As the relationship between GA and folate was not linear, alternative specifications were tested, including quadratic and spline terms; however, considering this variable as a category (i.e. by trimester) provided the best model fit.

False discovery rate adjustment using the Benjamini–Hochberg procedure [16] was applied to account for multiple hypothesis testing of the main predictors.

We tested for an interaction between gravidity and trimester because folate requirements are highest during the first trimester due to the formation of the neural tube, and multiparous women may enter pregnancy with lower folate stores than primigravidae. Other interaction terms between variables of interest were not considered because they were not supported by a biological hypothesis. For this subanalysis, we grouped women with >3 pregnancies together, as there were very few women with 4 or more pregnancies in their second trimester.

### Sensitivity analysis

We conducted 2 sensitivity analyses. First, because high inflammation in pregnant women can reflect a variety of conditions likely to impact their diet, health, and micronutrient status, we repeated the linear regression restricted to participants with a value of CRP <5 mg/L. Second, as 15 observations of serum folate were at the upper limit of detection of the assay (45.4 nmol/L), we used a right-censored Tobit regression (censoring point = 45.4 nmol/L) to handle observations at the upper detection limit.

All analyses were performed in R and R Studio Version 2024.4.2.764 [17] using the following packages: dplyr [18] and tidyr [19] for data handling, ggplot2 [18] for data visualization, censReg [20] for the sensitivity analysis with censored data, car [21] and splines [22] to check the linear model assumptions.

## Results

Of 450 women invited to participate in the study, 422 (93.8%) were enrolled, and 404 (95.7% of enrolled) had serum folate measured (Figure 1). The level of education was missing for 7 women, and the use of supplements was not reported for 1 woman. All other variables were available for all women, and this low level of missing value is not expected to have affected the analyses. Participants predominantly resided in urban settings, mainly in Addis Ababa or in the neighboring Oromia region. Most women were in the first trimester (85%), and the mean GA was 11 wk. Thirty percent of women were primigravidae.

A total of 61% of women had serum folate concentrations below levels estimated to be protective against NTDs. Mean dietary diversity was 3.8 (SD 0.9) of 10 food groups (Table 1). No women reported smoking or using any common medication known to alter folate metabolism.

**TABLE 1:** Sociodemographic characteristics of participants

	Mean (SD), median (IQR) or *N* (percentage)	Number of missing values
Maternal age (y)	26.4 (4.4)	0
Level of education		
No formal education	6 (2%)	7
Primary school	162 (40%)	
Secondary school	192 (48%)	
Higher education and above	37 (10%)	
Residence		
Urban	371 (92%)	0
Rural	33 (8%)	
Region		
Addis Ababa	310 (77%)	0
Oromiya	94 (23%)	
Religion		
Muslim	206 (51%)	0
Orthodox	172 (43%)	
Protestant	26 (6%)	
Occupation		
Government employee	20 (5%)	0
Private employee	52 (13%)	
Privately owned business	36 (9%)	
Housewife	232 (57%)	
No occupation	63 (16%)	
Other	1 (<1%)	
Trimester status		
First trimester	343 (85%)	0
Second trimester	61 (15%)	
Marital status		
Married	401 (99%)	0
Single or widowed	3 (1%)	
Gravidity (including the current pregnancy)		
1	121 (30%)	0
2	110 (27%)	
3	109 (27%)	
4	54 (13%)	
≥5	10 (3%)	
BMI (kg/m^2^)	24 (1.0)	0
Serum folate (nmol/L)	22.8 (9.2)	
C-reactive protein (mg/L)	1.6 (0.8, 4.0)	0
Serum folate <24.3 nmol/L)	247 (61%)	0
Mean dietary diversity score	3.8 (0.9)	0
Use of nutritional supplements^1^ preconception	11 (3%)	1
Use of nutritional supplements^1^ in early pregnancy	13 (3%)	1

The median and IQR are presented for C-reactive protein due to its skewed distribution.

1 All the supplements consumed preconception were iron–folic acid supplements, and the women who reported taking the supplements reported taking an entire box for a month, so the equivalent of 30 supplements.

### Univariate analysis

In unadjusted linear regression models, several maternal and socioeconomic factors were associated with serum folate concentrations (Table 2). Higher gravidity was strongly associated with lower serum folate, with a graded decrease from –3.0 nmol/L among women with 2 pregnancies to –16.7 nmol/L among those with 5 pregnancies compared with primigravidae (*P* < 0.001). Serum folate concentrations were higher in the second trimester compared with the first trimester (*β* = 8.3 nmol/L; *P* < 0.001). Increasing maternal age, higher BMI, urban residence, and higher household income were each inversely associated with serum folate. Folate concentrations were higher among Orthodox women than Muslim women.

**TABLE 2:** Unadjusted association between serum folate and potential predictors

	Unadjusted *β* (95% CI)	*P* value
Predictor		
Proximal determinants		
Food intake		
Dietary diversity score	0.8 (–0.2, 1.8)	0.14
Consumption of folate-rich foods	–0.7 (–2.1, 0.6)	0.27
Use of supplements		
Prepregnancy supplementation	–1.0 (–6.5, 4.6)	0.74
Early pregnancy supplementation	–0.7 (–5.8, 4.4)	0.79
Physiological factors		
Gravidity	Ref: primigravida	
2 pregnancies	–3.0 (–5.8, –0.1)	0.04
3 pregnancies	–8.0 (–10.6, –4.9)	<0.001
4 pregnancies	–13·3 (–16·8, –9·7)	<0.001
5 pregnancies	–16.7 (–23.8, –9.6)	<0.001
BMI	–3.2 (–4.0, –2.4)	<0·001
Pregnancy trimester	Ref: first trimester	
Second trimester	8.3 (5.9, 10.7)	<0.001
Maternal age (in y)	–1.0 (–1.1, –0.8)	<0.001
Underlying determinants		
Residence	Ref: rural	
Urban	–8.3 (–11.4, –5.1)	<0.001
Income	Ref: poorest	
Poorer	–4.9 (–7.7, –2.2)	0.005
Middle	–3.5 (–6.2, –0.7)	0.11
Richer	–3.2 (–6.0, –0.4)	0.16
Richest	–6.2 (–9.0, –3.4)	<0.001
Education	Ref: no formal education	
Primary school	–4.7 (–12.0, 2.7)	0.7
Secondary school	–1.0 (–8.3, 6.3)	0.9
Higher education	1.3 (–6.4, 9.1)	0·9
Religion	Ref: Muslim	
Orthodox	2.8 (1.0, 4.7)	0.008
Protestant	3.5 (–0.2, 7.2)	0.15
Adjustment factors for the serum folate biomarker		
CRP (log)	0.6 (–0.5, 1.6)	0.28
AGP (log)	–0.3 (–2.5, 1.9)	0.79

Abbreviations: AGP, α-1-acid glycoprotein; CI, confidence interval; CRP, C-reactive protein.

Dietary indicators, including dietary diversity score and reported consumption of folate-rich foods, were not significantly associated with serum folate. Preconception and early pregnancy supplement use was reported by 3% of women only, and was not associated with serum folate concentrations. Biomarkers of inflammation (CRP and AGP) were not associated with serum folate in unadjusted analyses (Table 2).

### Multivariate model

Before model fitting, collinearity diagnostics were performed. Because maternal age and gravidity were highly correlated, maternal age was excluded to avoid collinearity. Household income, level of education, and occupation were correlated; only income was retained as the indicator of socioeconomic position to prioritize variables with no missing values. Preconception supplement use was selected to represent supplement exposure.

In the multivariable linear regression model adjusting for dietary, socioeconomic, and inflammatory factors, higher gravidity remained independently associated with lower serum folate concentrations (Table 3). Compared with primigravidae, serum folate was lower by –2.2 nmol/L among women with 2 pregnancies (*P* = 0.04), –6.2 nmol/L among those with 3 pregnancies (*P* < 0.001), –11.2 nmol/L among those with 4 pregnancies (*P* < 0.001), and –13.7 nmol/L among those with 5 pregnancies (*P* < 0.001) (Table 3).

**TABLE 3:** Association between serum folate and potential predictors, adjusted for dietary, socioeconomic, and inflammatory factors

	Adjusted *β* (95% CI)	*P* value
Predictor		
Proximal determinants		
Food consumption		
Frequency of consumption of folate-rich foods	–0.89 (–2.0, 0.3)	0.13
Dietary diversity score	0.84 (–0.07, 1.8)	0.07
Use of supplement		
Consumption of supplement preconception	Ref: no consumption	
Consumption	–2.8 (–7.6, 1.9)	0.24
Physiological factors		
Gravidity	Ref: primigravida	
2 pregnancies	–2.2 (–4.2, –0.1)	0.04
3 pregnancies	–6.2 (–8.6, –3.8)	<0.001
4 pregnancies	–11.2 (–14.4, –8.0)	<0.001
5 pregnancies	–13.7 (–19.8, –7.6)	<0.001
Current BMI	–0.29 (–1.3, 0.7)	0.6
Trimester	Ref: trimester 1	
Trimester 2	3.9 (1.3, 6.4)	0.003
Underlying determinants		
Income quintile	Ref: poorest	
Poorer	–1.6 (–4.0, 0.9)	0.22
Middle	–1.7 (–4.1, 0.7)	0.17
Richer	–1.6 (–4.1, 0.8)	0.19
Richest	–2.5 (–5.0, –0.06)	0.04
Residence	Ref: rural	
Urban	–1.9 (–5.1, 1.3)	0.25
Religion	Ref: Muslim	
Orthodox	0.33 (–1.3, 2.0)	0.7
Protestant	0.06 (–3.3, 3.4)	0.97
Adjustment factors for serum folate biomarker		
CRP (log)	0.94 (–0.1, 2.0)	0.08
AGP (log)	–6.0 (–12.6, 0.7)	0.08

Abbreviations: AGP, α-1-acid glycoprotein; CI, confidence interval; CRP, C-reactive protein.

Serum folate concentrations were higher among women in the second trimester than those in the first trimester (*β* = 3.90 nmol/L; *P* = 0.003). Neither dietary diversity score nor consumption of folate-rich foods was associated with serum folate after adjustment. Preconception supplement use was not associated with serum folate. Serum folate was lower among women in the richest income quintile compared with the poorest (*β* = –2.5; *P* = 0.04), whereas there was no significant difference for women in other income quintiles. The association between religion and folate concentrations did not persist in the fully adjusted model.

### Assumptions of the linear model

The model fitting was considered adequate based on the linear relationship between variables, the normality of residuals, and the absence of collinearity among variables. Some heteroscedasticity was detected, as the variance of residuals was higher in higher serum folate concentrations. Because our analysis was focused on the determinants of low serum folate, no statistical correction to this heteroscedasticity was applied in the model. We acknowledge that other factors (including nutritional factors that our analysis did not capture) could influence the variability of serum folate at higher values.

### Interaction between gravidity and trimester

**FIGURE 2:**
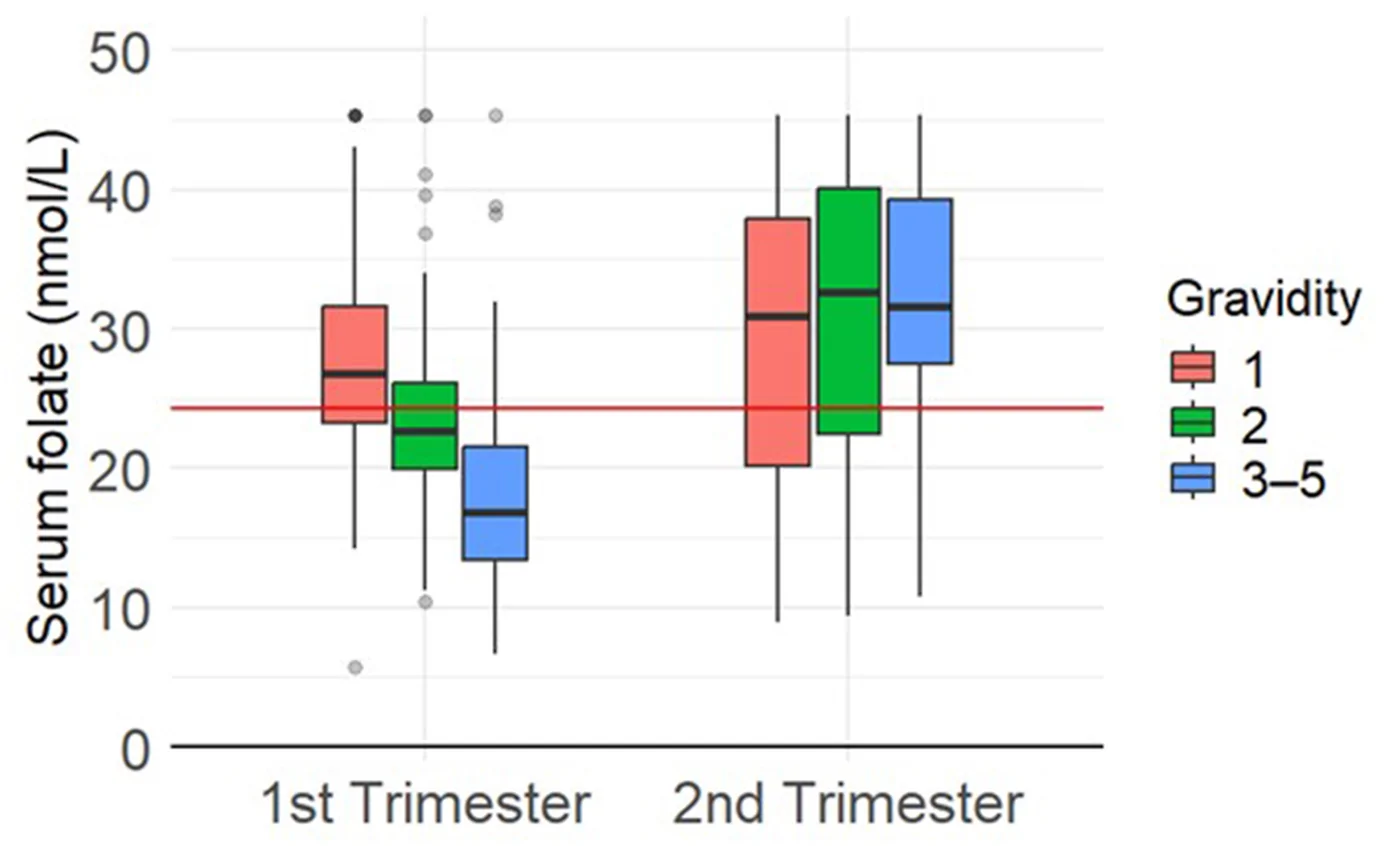
Maternal serum folate concentration by trimester and gravidity. Boxplots show serum folate concentrations by gravidity and trimester (median, IQR, whiskers = 1.5 × IQR). Gravidity categories represent the number of previous pregnancies (1, 2, and 3–5). Sample sizes were as follows: 1st trimester-gravidity 1 (*n =* 87), 2 (*n =* 91), 3–5 (*n =* 165); 2nd trimester-gravidity 1 (*n =* 34), 2 (*n =* 19), 3–5 (*n =* 8). The red line indicates the threshold for increased risk of neural tube defects affected pregnancy (24.3 nmol/L). Associations were confirmed in adjusted linear regression models (Table 3).

We fitted an interaction term between gravidity and trimester to assess whether the association between gravidity and serum folate differed by pregnancy stage. The interaction was statistically significant (*P* < 0.05), indicating that the effect of gravidity on serum folate varied between the first and second trimesters (Figure 2).

### Sensitivity analysis

Excluding women with elevated inflammation (CRP >5 mg/L; *n* = 78) did not change the direction or magnitude of the associations observed in the adjusted model. The inverse association between gravidity and serum folate and the positive association with second-trimester status remained statistically significant.

Similarly, results from the Tobit regression model, which accounted for right-censoring due to the upper detection limit of the folate assay, were consistent with those from the adjusted linear regression model. These findings indicate that the small number of samples with concentrations above the limit of detection did not bias the result.

## Discussion

### Main findings

We found that 61% of pregnant women attending their first ANC visit in Addis Ababa had serum folate concentrations below levels estimated to be protective against NTDs. This prevalence is higher than previously reported in a hospital-based study in Addis Ababa and is consistent with national values of folate deficiency reported in the 2015 national micronutrient survey. Furthermore, our study provides novel empirical evidence in an LMIC context that repeated pregnancies impose cumulative metabolic demands, depleting maternal folate stores and substantially increasing NTD risk. Although we did not assess birth outcomes, the strong dose–response association between gravidity and low serum folate concentrations observed during early pregnancy places multiparous women at increased risk of NTD-affected pregnancies, given that most fall below folate concentrations associated with optimal NTD prevention.

Gravidity and trimester were the strongest predictors of folate status: multiparous women had significantly lower serum folate concentrations in the first trimester than primigravid women. Several elements of Bradford Hill’s causality framework [23] support the interpretation that higher gravidity contributes to lower folate status in this population. First, temporality: gravidity necessarily precedes the current pregnancy and the serum folate measurement. Second, we observed a strong dose–response gradient that persisted after adjustment for socioeconomic, dietary, and inflammatory factors. Third, there is a strong biological plausibility. Each pregnancy and lactation period increases folate requirements for fetal growth, expansion of maternal tissues, and breast milk production. If dietary folate intake and supplement coverage are low, and interpregnancy intervals are short, maternal folate stores are unlikely to be fully restored before the next conception [24], leading to cumulative depletion. This mechanism is consistent with the maternal depletion framework, whereby nutrient stores mobilized during pregnancy and lactation are not fully restored before subsequent conception if dietary intake or supplementation is inadequate [25,26].

Fourth, our findings are consistent with the wider literature. Longitudinal studies have shown that circulating folate concentrations decline during pregnancy and may remain depressed for several months postpartum if not replenished [26]. Studies from high-income countries have reported lower folate concentrations among multiparous compared with primigravid women [27], although in such contexts, interpretation was complicated by high use of folic acid supplements.

Fifth, there is an analogy with other outcomes along the same causal pathway. Depletion in maternal nutritional status not only compromises maternal health but also heightens risk of adverse fetal outcomes, including NTDs. Higher birth order has been associated with increased risk of spina bifida, and this pattern has been interpreted in light of possible folate depletion [28]. Drawing on data from the National Birth Defects Prevention Study in the United States, Petersen et al. [29] reported a trend for higher birth defects following short interpregnancy intervals, although these trends did not reach statistical significance.

We found that serum folate concentrations were higher in the second trimester than the first trimester, which, to our knowledge, has not been reported in LMIC contexts previously. Similar trimester-related increases have been reported in the United States NHANES [11] and other cohort studies such as the Canadian Alberta Pregnancy Outcomes and Nutrition cohort [30], and a Belgian cohort [12] often attributed to increasing adherence to antenatal supplements later in pregnancy. However, because supplement use was rare in our sample, this finding may instead reflect the very high demand for folate during the first trimester, leading to rapid depletion of status, which is later partly replenished through usual dietary intake. Alternative explanations, including physiological changes in plasma volume or unmeasured changes in diet, are also possible; however, none contradict the interpretation that folate status is low due to metabolic demands in the early gestational period.

In unadjusted analyses, serum folate concentrations were higher among Orthodox women compared with Muslim women. However, this association did not persist in the fully adjusted model. Muslim women in this cohort had higher gravidity on average, and the attenuation of the association after adjustment suggests confounding by reproductive history rather than an independent effect of religion.

Dietary indicators, including dietary diversity score and consumption of folate-rich foods, were not associated with folate status. The lack of observed association between known dietary sources of folate and maternal folate status was unexpected; however, there was low heterogeneity in diets among participants, and the dietary indicator might be too crude to discern differences in folate intake.

### Policy relevance

In Ethiopia, maternal undernutrition remains a key contributor to adverse pregnancy outcomes. Our findings suggest that many women in Ethiopia enter pregnancy with inadequate folate status, even in urban areas among populations attending health facilities. Concerningly, other studies demonstrate that folate deficiency among adult women is even more widespread in several regions outside of Addis Ababa [6], whereas in Eastern Ethiopia, higher rates of NTDs were observed among children born to mothers with no ANC compared with those who received ANC [4].

Low serum folate values among pregnant women underscore the need for effective interventions targeting the preconception period. Ethiopia has introduced national preconception care guidelines [31], which acknowledge the need for folic acid supplementation, aligning with WHO recommendations. Our findings emphasize the public health relevance of this policy and provide timely evidence to support its operationalization, for example, by strengthening outreach to multiparous women. Furthermore, our findings suggest that extending the current iron–folic acid supplementation recommendation into the postpartum period may deliver benefits for subsequent pregnancies by aiding the replenishment of maternal folate stores.

Folate status may also be improved at the population level through large-scale food fortification. In 2024, Ethiopia launched a compulsory National Food Fortification Program, led by the Ethiopian Food and Drug Authority, mandating fortification of domestically produced and imported wheat flour with key micronutrients, including folic acid. Although implementation is underway across major flour-milling industries, early assessments highlight variable compliance and challenges in regulatory enforcement. Moreover, disparities in wheat flour consumption, particularly among rural and low-income households, may limit the population-wide impact of flour fortification. Research is ongoing to determine the feasibility and effectiveness of folic acid-fortified salt, which is more widely consumed than wheat flour in Ethiopia [32]. Additionally, recent research points to the importance of diversified agri-food systems to support adequate population folate status in Ethiopia [6].

### Strengths and limitations

This study has several strengths. The study recruited women early in pregnancy, providing a unique insight into first-trimester folate status in an LMIC setting. The mean GA was 11 wk, indicating that most women attended their first ANC visit toward the end of the first trimester. This relatively early attendance may reflect increasing awareness of early pregnancy care, improved availability of maternal health services at public health centers, and community-level health education by health extension workers [33]. In Ethiopia, early ANC initiation can also be driven by free ANC services, concerns about maternal nutrition, previous adverse pregnancy outcomes, and increased health-seeking behavior associated with educational access [34].

The low prevalence of supplement use enabled assessment of folate status unconfounded by supplemental folic acid intake, in contrast with most evidence from high-income settings. We were therefore able to clearly identify gravidity and trimester effects that had previously been hypothesized based on modeling studies or observed, but with limited statistical certainty in high-income country contexts.

The study followed a robust design, with a comprehensive collection of known determinants of folate status (e.g., supplement use, folate-rich food consumption, medications known to alter folate metabolism, and other maternal conditions), and robust statistical methods (e.g., exposures defined a priori, and adjustment for multiple hypothesis testing).

However, some limitations should be considered. Dietary data were based on short-term recall and may not reflect long-term intake. Serum folate reflects recent intake and does not fully capture long-term folate status; measurement of red blood cell folate would have strengthened interpretation. Serum folate was measured using a folate-binding protein-based immunoassay rather than reference standard microbiological or liquid chromatography-tandem mass spectrometry methods (LC-MS/MS) methods [35], which may underestimate folate concentrations, particularly at higher values. However, any underestimation of folate concentrations related to the assay is unlikely to have biased the association between serum folate and the number of prior pregnancies, as measurement error would be expected to be similar across gravidity groups.

Systemic inflammation may influence folate metabolism directly or reflect underlying conditions that impair nutrient absorption [36]. However, in this study, neither CRP nor AGP was independently associated with serum folate concentrations, and exclusion of women with elevated CRP did not alter the results. This suggests that inflammation is unlikely to be a major driver of folate depletion in this cohort, and does not explain the strong inverse association between gravidity and folate status.

Finally, the study was conducted in urban health centers in Addis Ababa, and studies are required in other settings to build evidence that is representative of the wider Ethiopian population.

In conclusion, low folate status was highly prevalent among pregnant women attending their first ANC visit in Addis Ababa, with more than half having serum folate concentrations below levels associated with optimal NTD prevention. Gravidity was strongly and inversely associated with folate status, and the interaction with trimester indicated that multiparous women are particularly vulnerable to folate depletion in early pregnancy, when folate is most critical for fetal neural development. Unlike findings from high-income settings, these results were not confounded by folic acid supplement use, which was very low in this population. Most women, therefore, entered pregnancy with inadequate folate reserves, highlighting a critical gap in current maternal nutrition strategies in Ethiopia. Improving folate status before conception—through expanded preconception care, increased access to periconception folic acid supplementation, and large-scale food fortification—should be prioritized to reduce risk of preventable NTDs.

## Author contributions

The authors’ responsibilities were as follows – HTM: conceptualization, data curation, formal analysis, investigation, methodology, project administration, writing - original draft, writing - review and editing; EJMJ: conceptualization, funding acquisition, project administration, methodology, supervision, writing - original draft, writing - review and editing; DG: project administration, supervision, funding acquisition, writing - review and editing; DB, TLG: investigation, resources, writing - review and editing; BS: methodology, resources, supervision, writing - review and editing; FS: conceptualization, data curation, formal analysis, software, methodology, supervision, writing - original draft, writing - review and editing; and all authors: read and approved the final version.

## Data availability

The data generated and analyzed during this study are not publicly available. Neither participant consent nor the approved ethics protocol included data sharing beyond the project, reflecting confidentiality concerns. Reasonable requests for access will be considered on a case-by-case basis, subject to a data-transfer agreement and approval by the ethics committee. Only de-identified data may be shared. Enquiries can be directed to the first author.

The data collection tools, R script for data analysis, as well as summary statistics not included in the manuscript (for example, per particular subgroup) are available on request.

## Funding

This work was supported by the Gates Foundation “GeoNutrition” project [INV-009129] and “Micronutrient Action Policy Support” project [INV-002855]. The conclusions and opinions expressed in this work are those of the authors alone and shall not be attributed to the Foundation. Under the grant conditions of the Foundation, a Creative Commons Attribution 4.0 License has already been assigned to the Author Accepted Manuscript version that might arise from this submission. The funder did not have any role in the design, implementation, analysis, and interpretation of the data.

## Conflict of interest

The authors report no conflicts of interest.

## Figures and Tables

**Fig. S1:**