# Supplementary material for: Prior Pregnancies Deplete Maternal Folate Status and Increase Risks of Neural Tube Defects in Addis Ababa, Ethiopia
Source: J Nutr. 2026 Jun 10;156(8):101666. doi: 10.1016/j.tjnut.2026.101666 (PMC13494172; doi:10.1016/j.tjnut.2026.101666)
Supplement: Supplementary file 1 [file mmc1.docx]

**Title**: Prior pregnancies deplete maternal folate status and increase risks of neural tube defects in Addis Ababa, Ethiopia.

**First author:** Hasset Tamirat Molla

**Supplementary text**

*Diet diversity score*

Ten predefined food groups were included: (1) grains, roots, and tubers; (2) pulses; (3) nuts and seeds; (4) dairy products; (5) meat, poultry, fish, and organ meats; (6) eggs; (7) green leafy vegetables and vitamin C–rich fruits; (8) vitamin A–rich vegetables; (9) other vegetables; and (10) other fruits [31]. Responses were coded as binary (consumed vs not consumed) for each food group per day. Group-level consumption was then averaged across the three recall days, such that a dietary diversity score of 1 indicated that a food group was consumed on all three days, 2/3 indicated two of the three days, and 1/3 indicated one of the three days. A diversity score was then created by adding the score for each food group. A score below 5 (out of 10) was considered low.^1^

*Food frequency questionnaire*

We used a food frequency questionnaire (FFQ) tailored to Ethiopian dietary patterns. The FFQ included a comprehensive list of commonly consumed food items grouped into 20 categories, such as cereals, legumes, dairy, meat, fruits, and vegetables. One category specifically captured foods naturally rich in folate (e.g., dark green leafy vegetables, and certain fruits). Participants reported the frequency of consumption for each food group over the previous week, enabling assessment of dietary diversity and intake of folate-rich foods.

**Grains**

Teff

Maize

Rice

Wheat

Sorghum

Barley

Amaranth grain

Pasta

Other foods made from grain

**Roots and tubers**

Plantains

Enset

White or yellow-sweet potato

Yam

White cassava

Other white starchy staples

**Pulses**

Chickpeas

Lentils

Peas

Cowpea

Bambara

Other beans, peas, or bean/pea products

**Nuts and seeds**

**Dark leafy greens**

Kale

Amaranth leaves

Cassava leaves

Sweet potato leaves

Other dark leafy greens, incl. foraged leaves

**Foods rich in folates**

Green soybean

Asparagus

Spinach

Broccoli

Avocado

Mango

Sweet corn

Orange

Other foods rich in folates

**Foods rich in Vitamin A**

Ripe mango

Guava

Ripe papaya

Cantaloupe

Other locally available orange fruits (specify)

100% fruit juice made from these orange fruits

Other foods rich in vitamin A

**Other vegetables**

Tomato

Onion

Eggplant

Green pepper

Cucumber

Cabbage

Other local vegetables

**Other fruits**

Ripe banana

Pineapple

Apple

Other local and/or wild fruits (specify)

100% fruit juice made from these other fruits

**Red palm oil**

**Milk and milk products**

Milk

Cheese

Yogurt

Other milk products, NOT incl. ice cream and butter

**Organ meat**

Liver

Other organ meat (kidney, heart, intestines)

**Meat and poultry**

Beef meat

Goat meat

Chicken meat

Lamb

Other flesh/poultry meat

**Fish and sea food**

Fish or dried fish, shellfish or seafood

**Eggs**

Chicken eggs

Other eggs

**Oil and fats**

Butter

Vegetable oil (fortified)

Vegetable oil (unfortified)

Other oil/fats for cooking and adding to food

**Savory and fried snacks**

Crisps, chips, French fries, fried dough and other fried foods

**Sweets and sweetened beverages**

Vitamin A-fortified sugar

Unfortified Sugar/honey (including use in beverages)

Cakes/Candies/chocolates/sweet biscuits

Sweetened juice, soda and other sugar-sweetened beverages

Other sweets

**Condiments and seasonings**

Iodized salt (if they do not know, can check container if available)

Non-iodized salt

Hot sauce, fish sauce, chilies, spices, herbs, tomato paste, flavor cubes, or other condiments

**Other beverages and foods**

Tea

Coffee

Clear broth

Alcohol

Any other foods

**Reference**

1.	FAO & FHI 360. *Minimum Dietary Diversity for Women: A Guide for Measurement. Rome: FAO.* (2016).
