# Supplementary material for: Prior Pregnancies Deplete Maternal Folate Status and Increase Risks of Neural Tube Defects in Addis Ababa, Ethiopia
Source: J Nutr. 2026 Jun 10;156(8):101666. doi: 10.1016/j.tjnut.2026.101666 (PMC13494172; doi:10.1016/j.tjnut.2026.101666)
Supplement: Supplementary file 2 [file mmc2.docx]

**Title**: Prior pregnancies deplete maternal folate status and increase risks of neural tube defects in Addis Ababa, Ethiopia.

**First author:** Hasset Tamirat Molla

**Supplementary Figure 1**: Conceptual framework illustrating the relationships between underlying and proximal determinants of maternal folate status. An asterisk (*) denotes that the variable (e.g., maternal age) or an appropriate proxy (e.g., inflammation assessed through inflammatory protein biomarkers) was measured within the study.
